# Supplementary material for: End Sequence Analysis Toolkit (ESAT) expands the extractable information from single-cell RNA-seq data
Source: Genome Res. 2016 Oct;26(10):1397–410. doi: 10.1101/gr.207902.116 (PMC5052061; doi:10.1101/gr.207902.116)
Supplement: Supplemental Material [file supp_gr.207902.116_Supp_Materials.tar.gz › ESAT-esat_v0.1/doc/html/interfacebroad_1_1pda_1_1seq_1_1segmentation_1_1_alignment_data_model-members.html]

ESAT: Member List


ESAT


- Main Page
- Related Pages
- Packages
- Classes
- Files

- Class List
- Class Index
- Class Hierarchy
- Class Members

All Classes Namespaces Files Functions Variables Enumerator Pages

- broad
- pda
- seq
- segmentation
- AlignmentDataModel

broad.pda.seq.segmentation.AlignmentDataModel Member List

This is the complete list of members for broad.pda.seq.segmentation.AlignmentDataModel, including all inherited members.

|  |  |  |
| --- | --- | --- |
| clearFullIntervalTreeAsAlignmentsCached(String chr) | broad.pda.seq.segmentation.AlignmentDataModel |  |
| getAnnotationOverlappingRegion(Annotation align) | broad.pda.seq.segmentation.AlignmentDataModel |  |
| getBasesCovered(Annotation record, int EF) | broad.pda.seq.segmentation.AlignmentDataModel |  |
| getBasesCoveredPerAlignment(Gene gene, IntervalTree< Alignment > tree, int EF) | broad.pda.seq.segmentation.AlignmentDataModel |  |
| getChromosomeLength(String chr) | broad.pda.seq.segmentation.AlignmentDataModel |  |
| getChromosomeLengths() | broad.pda.seq.segmentation.AlignmentDataModel |  |
| getChromosomeReadIterator(String chromosome) | broad.pda.seq.segmentation.AlignmentDataModel |  |
| getChunkEnd() | broad.pda.seq.segmentation.AlignmentDataModel |  |
| getChunkStart() | broad.pda.seq.segmentation.AlignmentDataModel |  |
| getCounts(String chr) | broad.pda.seq.segmentation.AlignmentDataModel |  |
| getCountsOfUniqueOverlappers(Annotation target, Annotation exclude, IntervalTree< Alignment > tree, int EF) | broad.pda.seq.segmentation.AlignmentDataModel |  |
| getCountsOfUniqueOverlappers(Annotation target, Gene exclude, IntervalTree< Alignment > tree, int EF) | broad.pda.seq.segmentation.AlignmentDataModel |  |
| getCountsOnBase(String chr, int index) | broad.pda.seq.segmentation.AlignmentDataModel |  |
| getCountsPerAlignment(Annotation first, int EF) | broad.pda.seq.segmentation.AlignmentDataModel |  |
| getCountsPerAlignment(Annotation align, IntervalTree< Alignment > tree, int EF) | broad.pda.seq.segmentation.AlignmentDataModel |  |
| getCountsPerAlignment(Gene gene, IntervalTree< Alignment > tree, int EF) | broad.pda.seq.segmentation.AlignmentDataModel |  |
| getCountsPerAlignment(Annotation[] Annotation, IntervalTree< Alignment > tree, int EF) | broad.pda.seq.segmentation.AlignmentDataModel |  |
| getCountsPerAlignment(Annotation[] Annotation, int EF) | broad.pda.seq.segmentation.AlignmentDataModel |  |
| getCountsPerAlignment(Annotation align, Map< String, IntervalTree< Annotation >> goodExonTree, IntervalTree< Alignment > tree, int extensionFactor) | broad.pda.seq.segmentation.AlignmentDataModel |  |
| getCountsPerAlignmentFullyContained(Gene gene, IntervalTree< Alignment > tree, int EF) | broad.pda.seq.segmentation.AlignmentDataModel |  |
| getCountsPerAnnotationtranded(Gene gene, IntervalTree< Alignment > tree, int EF, String orientation) | broad.pda.seq.segmentation.AlignmentDataModel |  |
| getCountsWithinExons(Annotation align, Iterator< Alignment > iter, int EF) | broad.pda.seq.segmentation.AlignmentDataModel |  |
| getCountsWithinExons(Annotation align, IntervalTree< Alignment > tree, int EF) | broad.pda.seq.segmentation.AlignmentDataModel |  |
| getExonAnnotationOverlappingRegion(Annotation align) | broad.pda.seq.segmentation.AlignmentDataModel |  |
| getFullIntervalTreeAsAnnotation(String chr) | broad.pda.seq.segmentation.AlignmentDataModel |  |
| getGeneCounts(Gene gene, int extensionFactor) | broad.pda.seq.segmentation.AlignmentDataModel |  |
| getIntervalTree(String chr, int start, int end) | broad.pda.seq.segmentation.AlignmentDataModel |  |
| getIntervalTreeCached(String chr, int start, int end) | broad.pda.seq.segmentation.AlignmentDataModel |  |
| getIntervalTreeTruncatedCached(String chromosome, int start, int end) | broad.pda.seq.segmentation.AlignmentDataModel |  |
| getMinimumMappingQuality() | broad.pda.seq.segmentation.AlignmentDataModel |  |
| getModelFilePath() | broad.pda.seq.segmentation.AlignmentDataModel |  |
| getNumberOfSplicedReads(Annotation align) | broad.pda.seq.segmentation.AlignmentDataModel |  |
| getOverlappingRegions(String chr) | broad.pda.seq.segmentation.AlignmentDataModel |  |
| getPeak(Gene gene, Gene startCodon, IntervalTree< Alignment > tree, int EF) | broad.pda.seq.segmentation.AlignmentDataModel |  |
| getReadIterator() | broad.pda.seq.segmentation.AlignmentDataModel |  |
| getReadIterator(Annotation region) | broad.pda.seq.segmentation.AlignmentDataModel |  |
| getScorePerAlignmentFromCache(Gene window, IntervalTree< Alignment > chunkAlignmentTree, int extensionFactor) | broad.pda.seq.segmentation.AlignmentDataModel |  |
| getSplicedReadExons(String chr) | broad.pda.seq.segmentation.AlignmentDataModel |  |
| getSplicedReads(Annotation align, int minIntronSize, int maxIntronSize) | broad.pda.seq.segmentation.AlignmentDataModel |  |
| getSplicedReads(Annotation region, Collection< Predicate< Annotation >> filters) | broad.pda.seq.segmentation.AlignmentDataModel |  |
| getSplicedReads(Annotation region) | broad.pda.seq.segmentation.AlignmentDataModel |  |
| getSplicedReads(Annotation align, final int minIntronSize, final int maxIntronSize, int minNumIntrons, Sequence chrSeq) | broad.pda.seq.segmentation.AlignmentDataModel |  |
| getSplicedReads(Annotation region, Collection< Predicate< Annotation >> filters, int minNumIntrons) | broad.pda.seq.segmentation.AlignmentDataModel |  |
| getSplicedReads(Annotation region, Collection< Predicate< Annotation >> filters, int minNumIntrons, Sequence chrSeq) | broad.pda.seq.segmentation.AlignmentDataModel |  |
| getSplicedReads(Annotation region, Predicate< Annotation > filter) | broad.pda.seq.segmentation.AlignmentDataModel |  |
| getSplicedReads(int minIntronSize, int maxIntronSize) | broad.pda.seq.segmentation.AlignmentDataModel |  |
| getSpliceWeightFactor() | broad.pda.seq.segmentation.AlignmentDataModel |  |
| getTotalNumberOfStrandedReads() | broad.pda.seq.segmentation.AlignmentDataModel |  |
| hasNextExon(String chr) | broad.pda.seq.segmentation.AlignmentDataModel |  |
| isNegativeStranded() | broad.pda.seq.segmentation.AlignmentDataModel |  |
| isPositiveStranded() | broad.pda.seq.segmentation.AlignmentDataModel |  |
| isStranded() | broad.pda.seq.segmentation.AlignmentDataModel |  |
| passes(Gene gene, IntervalTree< Alignment > tree, int EF, double cutoff) | broad.pda.seq.segmentation.AlignmentDataModel |  |
| passesCutoff(Annotation align, IntervalTree< Alignment > tree, int EF, double threshold) | broad.pda.seq.segmentation.AlignmentDataModel |  |
| resetTreeCache() | broad.pda.seq.segmentation.AlignmentDataModel |  |
| resetTreeCache(String chr) | broad.pda.seq.segmentation.AlignmentDataModel |  |
| restartIterator() | broad.pda.seq.segmentation.AlignmentDataModel |  |
| setChunkSize(int chunkSize) | broad.pda.seq.segmentation.AlignmentDataModel |  |
| setExtensionFactor(int factor) | broad.pda.seq.segmentation.AlignmentDataModel |  |
| setFirstRead() | broad.pda.seq.segmentation.AlignmentDataModel |  |
| setMinimumMappingQuality(double minimumMapQuality) | broad.pda.seq.segmentation.AlignmentDataModel |  |
| setNegativeStranded() | broad.pda.seq.segmentation.AlignmentDataModel |  |
| setNormalizationStrategy(ReadCountNormalizer normalizer) | broad.pda.seq.segmentation.AlignmentDataModel |  |
| setPositiveStranded() | broad.pda.seq.segmentation.AlignmentDataModel |  |
| setSecondRead() | broad.pda.seq.segmentation.AlignmentDataModel |  |
| unsetStranded() | broad.pda.seq.segmentation.AlignmentDataModel |  |
| updateGeneByFirstCounts(Gene gene, IntervalTree< Alignment > tree, int EF) | broad.pda.seq.segmentation.AlignmentDataModel |  |


---

Generated on Fri Aug 15 2014 15:11:04 for ESAT by  

 1.8.7
